# Promoting Participation in African High Schools: A Scoping Review

**DOI:** 10.1155/oti/1536306

**Published:** 2026-07-15

**Authors:** Lomarie Thesnaar, Monique De Wit, Fasloen Adams, Nicola Ann Plastow

**Affiliations:** ^1^ The Division of Occupational Therapy, Stellenbosch University, Bellville, Western Cape, South Africa, sun.ac.za

**Keywords:** adolescents, framework analysis, interventions, occupational therapy, participation, schools

## Abstract

Learning is the primary occupation of adolescents in high schools, although they engage in a much wider range of occupations at school. Recent systematic and scoping reviews from high‐income countries (HICs), classified by the World Bank, with the United States as the most prominent, provide evidence to support the role of occupational therapy in school participation for adolescents with developmental, health, and environmental barriers. However, participation for high school learners in the African context differs from that of learners from an HIC context, due to the unique challenges faced by learners in Africa. These challenges include poverty, educational challenges (e.g., early school dropout and teacher shortfall), infrastructure and resource shortages, cultural expectations and inequalities (e.g., family and gender norms), conflict, and instability. A scoping review was conducted to map interventions that promote participation in schools internationally and to evaluate their suitability for the African context. The Preferred Reporting Items for Scoping Reviews and Meta‐Analyses (PRISMA) extension guidelines were used to guide this review. Sources about the nature of school‐based interventions for adolescents (12–18 years) that indicated a clear link between the intervention and participation in schools, published in English (with no restriction on years) and peer‐reviewed, were included. A total of 2689 studies (excluding duplicates) were identified, and 15 were included. Framework analysis was used to evaluate the suitability of interventions, including the characteristics of the evidence, community‐based practice, practice approaches, sustainability, cultural norms, and spirituality. Four interventions were identified as suitable for the African context: a recovery program for adolescents with substance use disorders, a social cognitive intervention to promote social skills in adolescents with autism, a transition readiness service to promote work participation, and coping strategies to promote adolescents′ mental health in schools. Recommendations were made to include spirituality when delivering interventions for adolescents in African schools.

## 1. Introduction

Education is the vehicle for economic growth and development in African countries [1]. Despite this, Africa is faced with unique challenges to education. School access remains the lowest in this region [2], with the completion rate for sub‐Saharan adolescents at 40% in lower secondary schools [3]. Additionally, 36.6% of these adolescents between 12 and 14 years and 57.8% between 15 and 17 years are not in educational settings [4]. Various challenges impede adolescents′ participation in African schools, with one of the main challenges being socioeconomic factors [5]. In Tanzania, socioeconomic factors such as illiterate families, negative attitudes toward learning, parental low income, and cultural aspects (e.g., early marriage or pregnancy) were listed as the main reasons for adolescents leaving school [6]. Additionally, conflict and war caused Ethiopian adolescents to leave school due to youth movements in wartime, being recruited to participate in wars, the demolition of the school infrastructure, poor ability to focus on education due to stress and trauma, and the loss of educational motivation [7]. Furthermore, disability or illness was the main reason (22.7%) for learners leaving school in South Africa, followed by poor academic achievement (21.2%) [8]. Within the rural areas in Africa, these challenges are even worse, which has a ripple effect on the quality of education being received [9–11].

Schools are seen as one of the main settings where occupational therapists can make a valuable contribution by promoting participation, well‐being, and inclusion [12]. This includes promoting participation in a wide variety of occupations, including learning, literacy, handwriting, transition services, social participation, assistive technology, life skills, making accommodations, and empowering and advocating [13–16]. Occupations in schools can refer to academic (e.g., mathematics, reading, and writing) as well as nonacademic activities (e.g., eating lunch, socializing with peers, and singing in a choir), which are both important for learners′ holistic participation in school [17]. Internationally, there is evidence showing that improved participation in schools could positively affect school performance, social participation, character development, community involvement, adolescent well‐being, and lower school dropout and delinquency [18–21].

In contrast to Western philosophies, which favor the medical model, individuality, and independence, African philosophies are centered around a dislike for individualism, a preference for a community‐based society, and spirituality [22, 23]. Many occupational choices made by adolescents are based on the values of interdependency, reciprocity, relatedness, and spirituality to one another within their community, and therefore, these ultimately shape their occupational choices and their participation in schools. Jansen‐van Vuuren et al. [19] conducted a scoping review on the role and scope of occupational therapy in Africa, where certain contextual considerations for practicing occupational therapy in various African contexts were identified. These contextual considerations consist of community‐based services, practice approaches, sustainability, cultural norms, and spirituality.

Occupational therapy practice in Africa should be community‐based, where services should be prioritized in communities, as this allows for client‐centered, occupation‐focused, culturally sensitive, and holistic practice. Many occupational therapists in Africa are currently using a population approach to ensure provision for the community as a whole by prioritizing African values of collectivism and interdependence with high‐income families and community networks [24–30].

Collaboration with community members such as families, health workers, and community leaders is an important occupational therapy role that ensures a population approach. For example, in Tanzania, collaboration between a local community‐based rehabilitation program and an international aid organization was used to fund children with disabilities as well as address challenges of poverty, drought, and malnutrition in the whole community and family through income‐generation initiatives [31]. Various authors emphasize occupational therapy group interventions rather than exclusively individual interventions, such as Ugandan HIV/AIDS income‐generation groups and South African pediatric interventions [32–34]. Community‐based approaches such as health promotion and disability prevention are also important elements that guide occupational therapy interventions in Africa [25, 26].

Ubuntu is also an important principle that guides sub‐Saharan African occupational therapy practice and theory, which values interdependence and relationships and where a person′s identity is characterized by their involvement in a community [19]. Many of the elements of Ubuntu (e.g., respect, spirituality, and reciprocity) align with occupational therapy theoretical concepts such as addressing societal needs while valuing humankind [35, 36].

Practice approaches, focusing on African‐specific models for planning assessments and interventions, should include spirituality (which provides meaning to people′s daily lives), cultural norms (e.g., gender and family roles), and community survival. These practices should be sustainable, despite the unavailability of resources, language barriers, cultural norms such as interdependence within families/communities, corruption, and unethical practices in health care. Occupational therapists should, therefore, be creative in finding cost‐effective solutions as well as using local resources to address challenges to school participation faced on the continent.

Supporting adolescents′ participation in schools is embedded within occupational therapy′s scope of practice; however, there remains a gap in current research on interventions to promote school participation that are directly transferable to the African context. A set of guidelines for occupational therapy practice for children and youth was released in 2020 by the American Occupational Therapy Association (AOTA), focusing on interventions for the United States and interventions for similar high‐income contexts, which are not suitable for resource‐sensitive contexts [37]. Furthermore, the guidelines focused largely on occupational therapy interventions that promote academic performance (learning) in schools, while less emphasis was placed on other equally significant areas of occupational participation, such as social participation, extracurricular activities, and health and well‐being.

A similar trend was found in the systematic review of Grajo et al. [38], which only included articles related to academic participation and based on three themes: interventions to support learning, literacy, and handwriting. Limited databases were also selected (OTseekr, MEDLINE, CINAHL, Cochrane databases, and PsycINFO), and only English articles were included in this particular review. Additionally, this review included interventions that were part of the occupational therapy scope of practice, irrespective of the fact that they were not conducted by occupational therapists, which might be problematic when applying these interventions in school‐based practice. The sample sizes of the included articles were also small, and there were limitations in random sampling and control groups. This review did not report on the locations of the interventions, and consequently, no implications could be made for practice in Africa [38]. Another scoping review of [15]) examined occupational therapy interventions in school settings for learners aged 3–16 years. Similarly, it was found that the majority of studies (*n* = 50) focused on interventions to improve academic performance, with fewer studies focusing on interventions related to participation (*n* = 16) and health and well‐being (*n* = 4). This scoping review only used scientific journals, without including grey literature that might also be applicable [15].

A more recent scoping review by Clopper et al. [13] included studies that described the role of occupational therapy in middle to high school settings by delivering interventions addressing social participation, transition services, fine motor and handwriting, assistive technology, literacy participation, and life skills. One of the problems with this review is its limited applicability to practice in Africa. For example, occupational therapy in the United States is guided by the AOTA practice framework. School‐based occupational therapy practice in the United States also differs from that in African countries since the focus lies mainly on developing interventions for the individual, in comparison to the occupational therapists in Africa′s focus on changing the broader community surrounding the individual [19]. Occupational therapists in the United States are placed by federal policies in all schools to help children with disabilities, whereas in many African countries, occupational therapy posts in public schools are incredibly limited. The number of occupational therapists per population in Africa is eight times lower than the number recommended by the WFOT [39]. This indicates that therapists practicing in Africa have less time to spend with a patient compared to therapists practicing in HICs, like the United States [40]. The reality is that the only option to support adolescents is currently through private practice or services based in faith‐based schools, which causes further financial strain on families caught up in the cycle of poverty. This resonates with the critique voiced by [23]) against the application of domains of occupation rooted in Western models of participation. Although Clopper et al. [13] provided some interventions to promote participation in schools, it was concluded that there was limited research on specific interventions for middle and high school settings and that occupational therapy′s role in these school settings was not clearly defined. In support of this finding, a large amount of research focuses on interventions supporting primary school learners (i.e., elementary or middle school learners), with little focus on specific interventions to support adolescents or high school learners [41]. This was evident in a mapping review on occupational therapy interventions from 11 African countries, which indicated that most research studies represented the field of pediatrics (children between the ages of 4 and 6 years compared to high school learners) [40].

Scoping reviews are useful to provide a broad overview of a topic by clarifying key concepts as well as identifying gaps in the evidence (The [42]). This scoping review is aimed at mapping the nature of school‐based occupational therapy interventions to promote the participation of adolescents and then evaluating their appropriateness for the African context. A scoping review as a methodology was chosen as it provides evidence that may inform school‐based practice in Africa, compared to a systematic review, where the results are used to answer a specific research question [43].

## 2. Materials and Methods

The proposed framework by Arksey and O′Malley, together with the enhancements suggested by Levac et al., was used to conduct this review [44, 45]. The Joanna Briggs Institute Reviewers′ Manual was followed as a guide to compile this scoping review, and the PRISMA extension for scoping reviews (PRISMA ScR) checklist with six stages was followed to guide the methods of this review [46]. These six stages included identifying the research questions, identifying relevant studies, selecting the studies, data charting, reporting, collating, summarizing the results, and consultation.

### 2.1. Search Strategy

The research question that directed this review was as follows: What is known about the nature of occupational therapy interventions, appropriate for Africa, to promote participation in schools for adolescents aged 12–18 years? With this question in mind, the search terms were developed in consultation with the research supervisors and the faculty librarian. The search terms were “Occupational Therapy” AND “School” or classroom AND adolescen ^∗^ or teen ^∗^. Studies were identified by choosing databases that include health science professions and the field of education that applies to the research question: EBSCOhost: CINAHL, MEDLINE, Academic Search Premier, African Wide Information, ERIC, and Teacher Reference Centre. An initial search was conducted in January 2023, followed by a final search in June 2023 using search terms across all the chosen databases. Additional studies, which might have been relevant to the research question but were not identified in the database search, were hand‐searched by looking through the reference lists of the final list of included articles. The initial search was undertaken in collaboration with all four authors to ensure that the search delivered studies applicable to the research question.

### 2.2. Selection Criteria

Sources about primary research on the nature of school‐based interventions for adolescents (12–18 years) that indicated a clear link between the intervention and participation in or at schools, published in English and peer‐reviewed (with no restriction on years), were included in the review.

### 2.3. Review Process

A total of 3399 studies were identified and stored on Rayyan, a review management software. Rayyan was used to keep track of the search history and to keep an audit trail of all decisions made throughout the whole process of the study. A first (LT) and second reviewer (GR) were used to screen the studies. After removing the duplicates, the reviewers screened the titles and abstracts of the studies to identify possible relevant articles. Uncertainties were coded as “maybe” and discussed between the reviewers. Both LT and GR screened the full‐text articles against the selection criteria and resolved conflicts through discussions. A third reviewer (MDW) resolved any remaining conflicts. The first reviewer screened the reference lists of the included articles to identify any other studies that might meet the eligibility criteria.

### 2.4. Data Extraction

A Microsoft Excel Spreadsheet was compiled by the first reviewer to chart the data. To enhance credibility, this sheet was reviewed by the whole research team. Additionally, interrater reliability was ensured by extracting data from the first 10 articles collectively, as the research team (e.g., supervisors and reviewers). The first reviewer extracted data on the year, author, sample size, method, country of publication, the study aims, research design, and demographic information of participants, as well as their diagnoses, type, place of school, and educational level. The Cochrane PROGRESS Plus health equity framework guided data extraction on the place of residence, culture, language, gender, education, socioeconomic status, and social capital [47]. Information about the level of evidence of the data was extracted by using the NHMRC levels of evidence, which include five key components: the evidence base (the number, level, and quality of studies), the consistency of the study results, the clinical impact of the findings (interventions), the generalizability of the findings, and the applicability of the findings [48].

Data about the interventions were extracted using the categories from the TIDieR checklist. These categories included the name, goal, materials, procedures, service provider, modes of delivery, location, and modification [49]. Additional categories about occupational therapy roles, change modalities, materials, procedures, frequency, duration, number of sessions, and clinical impact were also included. Furthermore, recommendations for clinical practice and future research were extracted from the studies.

### 2.5. Data Analysis

Framework analysis is a type of thematic analysis that uses an organized structure of themes (the framework), developed inductively or deductively, to perform a cross‐sectional analysis of data [50]. The researchers followed the five steps of framework analysis proposed by Ritchie and Spencer [51]: In Step 1, data familiarization was achieved through the process of data extraction. In Step 2, our predetermined framework was based on the five contextual considerations of culturally relevant practice for Africa, proposed by Jansen‐van Vuuren et al. [19], and the characteristics of the evidence. These five contextual considerations included community‐based practice, practice approaches, sustainability, cultural norms, and spirituality. Occupational therapists in Africa have a unique role to play in health care practice, and therefore, occupational therapy interventions should be tailored to suit the specific cultural context in which they are delivered to ensure evidence‐based practice. Since we were interested in which domains of participation were a focus for school‐based practice, we used the domains from an African scope of practice document [52] to classify interventions (in this way, the unique cultural norms of Africa in health care were utilized to identify a framework).

In Step 3 (indexing), the extracted data was transferred from the Excel sheet into the framework. In Step 4 (charting and mapping), we considered which interventions identified in the review provided evidence for suitability for Africa across the factors. In Step 5 (interpretation), the researchers inductively interpreted the contextual relevance of each intervention and how it addresses the domains of participation. Consequently, we selected the interventions that showed the most promise for cultural adaptation to the African context.

A diagram shows the identification of the framework and creation of themes, subthemes, and components in Table 1.

**Table 1:** The proposed framework for evaluating interventions for suitability for Africa.

Characteristics of evidence	Community‐based	Practice approaches	Sustainability	Cultural norms for participation	Spirituality
Date	Location	TIDieR checklist	Equipment	Gender and family roles	Seven dimensions of spirituality [53]: Suffering, becoming, meaning, being, centeredness, connectedness, and transcendence
Journal	Members of the community	Name of the intervention	Duration/frequency	Language	
NHMRC level of evidence	PROGRESS‐Plus factors: Age, gender, diagnosis, type of school, socioeconomic status, and religion	Service provider	Group/individual	African humanism (beliefs and values)	
Study design		Duration/frequency	Prioritizing resources	HPCSA scope for practicing in Africa: Personal and community living (PCL), work, leisure, social interaction, play, and Learning	
Sample size		Group/individual			
Country published		Health condition			

## 3. Results

### 3.1. Study Selection

**Figure 1:**
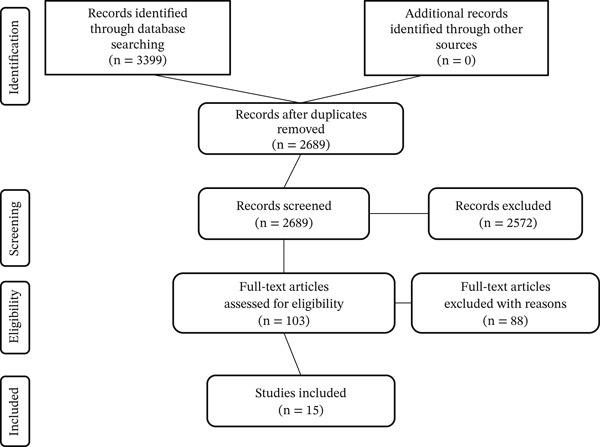
PRISMA flow diagram.

Through database searching, 3399 studies were identified, and 2689 studies remained after removing duplicates. The following phase involved the screening of the titles and the abstracts, where 2586 studies were excluded. A total of 103 studies were eligible for full‐text review. During the full‐text screening, 88 articles were excluded. The main reasons for exclusion were that the studies fell outside the age bracket (12–18 years), the intervention was not school‐based, and the intervention was not conducted by an occupational therapist. A final number of 15 articles was identified for this scoping review. The process of selecting studies is presented in Figure 1.

### 3.2. Characteristics of the Evidence

A total of 15 studies on interventions that promoted the participation of adolescents in schools were included (see studies summarized in Table 2). The publication dates ranged from 1987 to 2022, with most of the studies (12) published in the last 10 years. Four studies were published in the *American Journal of Occupational Therapy* (AJOT) [54–57]. Two studies were published in the *Journal for Occupational Therapy, Schools, and Early Intervention* [58, 59], and nine studies were published in different journals. None of the studies identified were conducted on the continent of Africa. More than 90% of the included studies were conducted in the United States or other HICs, while the other 10% of the included studies were conducted in low–middle‐income countries such as India and Hong Kong (two studies did not report on the country). The NHMRC levels of evidence found no Level I or Level II studies. Eight Level III studies (which mostly included case–control designs) were found, and seven Level IV studies were found (which mostly included case reports).

**Table 2:** Table of evidence.

First author and publication year	Name of the intervention	Country low/high income	Diagnosis, gender, age	Community‐based: Type of school and social capital	Practice approaches (individual/group)	Sustainability		Participation within cultural norms (learning/social/work/leisure/PCL)	Spirituality
						Frequency	Equipment		Dimensions of spirituality
Ali & Nagar [60]	Relaxation and desensitization	India, low income	—, 12–17 years, male and female	Public school	Individual	2× *p*/*w*	—	Social	—
Cheung et al. [58]	Social cognitive intervention program	Hong Kong, high income	ASD, 12–14 years, male	Public school	Group	1× *p*/*w*	—	Social	—
[61]	Program promoting social skills	—	ASD, 17 years, male	Public school, group home member, and teacher	Group	—	Equipment	Social	—
Gutman et al. [62]	Motor‐based social skills intervention	United States, high income	ASD, 15 years, male	Special needs school	Group	1× *p*/*w*	Equipment	Social	—
Hartwick & Yuen [54]	The compensatory counting system	United States, high income	Intellectual disability, 13 years, male	Public school	Individual	1× *p*/*d*	Equipment	Learning	—
Kinnealey et al. [55]	Installation of sound‐absorbing walls and halogen lighting in a classroom	United States, high income	ASD, 13–20 years, male	Private school	Group	—	Equipment		—
Larrington [63]	Sensory integration–based program	United States, high income	Multiple diagnoses, 15 years, male	Special needs school, group home staff, mother, and teacher	Individual	1× *p*/*w*	Equipment	Learning, leisure, and PCL	—
Merz et al. [64]	Group transition program	United States, high income	Multiple diagnoses, 13–20 years, male and female	Public school	Group	—	—	Work	—
Pierce et al. [65]	Transition readiness interventions	United States, high income	Multiple diagnoses, 14–16 years, —	Public school and peer group	Group	1× *p*/*w*	Equipment	Work	—
Pierce et al. [59]	Transition readiness services	United States, high income	Multiple diagnoses, 14–16 years, male and female	Public school, parents, staff, and community	Group	1× *p*/*w*	Equipment	Work	—
Selanikyo et al. [66]	The Collaborative Consultation for Participation of Students With Intellectual and Developmental Disabilities (Co‐PID)	Israel, high income	Multiple diagnoses, 8–20 years, male and female	Special needs school	Group	2× *p*/*m*	—	Learning	—
Selanikyo et al. [56]	The Collaborative Consultation for Participation of Students With Intellectual and Developmental Disabilities (Co‐PID)	Israel, high income	Multiple diagnoses, 7–20 years, male and female	Special needs school	Group	2× *p*/*m*	—	Learning	—
Thaler Souza et al. [67]	Program promoting mental health and well‐being	Brazil, low income	Mental health issues, 15–18 years, —	Public school	Group	—	Equipment	Social	—
Tomchek et al. [57]	Group‐based social skills training	—, —	ASD, 17 years, male	Public school	Individual	—	Equipment	Social and PCL	—
Wilburn et al. [68]	Strategies To Occupations in Recovering Youth (STORY) program	United States, high income	Substance use disorder, 18 years, male and female	Substance use recovery school	Group	1× *p*/*w*	—	Social	—

Abbreviations: —, not reported; ASD, autism spectrum disorder; PCL, personal and community living.

### 3.3. Community‐Based Practice

All the included studies provided occupational therapy in a school setting. Most studies did not report on the location of the school. Two schools were in an urban area [64, 68], and one school was located in both a rural and an urban area [65]. Furthermore, interventions were conducted in nine public schools, four special needs schools, one substance use recovery school, and one private school. Only three studies reported on the socioeconomic status of the school, which were middle [62], low–middle [66], and low, middle, and high [59]. Communities refer to groups of people, which include learners. The learners in the included studies were aged 12–18 years. The majority of studies reported on males [58, 61, 62, 54, 55, 63, 57]. Other studies reported on males and females together [60, 64, 59, 66, 56, 68]. Two studies did not report on gender [65, 67]. None of the studies reported on learners′ religion. School‐based interventions targeted learners with a wide variety of diagnoses. Learners diagnosed with multiple conditions (e.g., learning, developmental, and intellectual disabilities) were mostly reported on.

Most interventions (especially related to work and academic participation) were aimed at learners with multiple conditions ([64, 65, 59, 66, 56]). Interventions that promoted social and academic participation were mainly aimed at learners with autism [58, 61, 62, 57].

### 3.4. Practice Approaches

Overall, the studies show that occupational therapy services were primarily delivered through group‐based interventions [58, 62, 64, 65, 59, 67, 68]. Most of the interventions were conducted in collaboration with other team members [58, 61, 63–65, 59, 66, 56, 67, 57, 68], with teachers identified as the most common team members for interventions delivered individually. Interdisciplinary teams (consisting of various members, e.g., parents, community collaborators, and teachers) worked in consultation with occupational therapists to deliver transition services [64, 65, 59].

Seven adolescent‐focused interventions used groups as their main intervention modality. In the Strategies To Occupations in Recovering Youth (STORY) program, the occupational therapist and community health educators, as cofacilitators, provided group sessions to learners in a substance use recovery high school on narrative interviewing, reflective listening, goal setting, identity exploration, and occupational engagement [68]. Two group‐based interventions aimed at youth diagnosed with autism were also identified. One intervention provided motor‐based social skills, which included role‐play methods where motor behaviors were linked with their cognitive and emotional meanings [62]. Similarly, the occupational therapist and another team member provided a 10‐week social–cognitive intervention program where core social skills were taught in each session [58]. A school‐based mental health intervention by an occupational therapist and a teacher guided adolescents by applying a five‐stage coping process: initial dynamics, risk and protective factors, psychic distress and coping strategies, and final dynamics and reception [67].

A further three studies, which focused on transition interventions, were also delivered in a group format. One study examined the effectiveness of a school‐based occupational therapy transition readiness intervention. In this program, a combination of occupation‐based interventions (e.g., prevocational exploration, life skills activities, and long‐term learner‐led projects) was delivered in peer groups as well as transition team meetings [65]. The second occupational therapy intervention focused on coping with the transition of learners with developmental and learning disabilities and involved communication with the interdisciplinary team about the transition plan for learners, as well as group intervention sessions with the following focus areas: prevocational skills, social skills, self‐determination, physical fitness, and sensory processing [64]. The third study reported on the effects of occupational therapy transition readiness services on learners with disabilities. The intervention included topics such as self‐determination, prevocational exploration, life skills, social and communication skills, organization skills, and collaborations with staff (e.g., through IEP team meetings), parents (through education), and the community (e.g., coordinated job tours and bridging between in‐school and on‐site work experience) [59]. All the transition interventions were delivered in a group.

Another group‐based intervention highlighted by the literature, the Collaborative Consultation for Participation of Students With Intellectual Disabilities Program (Co‐PID), was conducted by occupational therapists and teachers through consultative meetings focusing on three aspects: communication, choosing, and initiating, which were vital for learners′ participation ([66, 56]).

Two studies targeted the environment and activity to intervene. The first occupational therapy intervention reported on modifying the classroom to enhance learning and attention of learners with severe communication disorders (e.g., autism). This modification included the installation of sound‐absorbing walls and halogen lighting [55]. Similarly, the second reported intervention, specifically for learners diagnosed with intellectual disability, focused on adapting counting activities. The latter intervention involved individual sessions with a learner [54].

Four interventions targeted adolescents individually. A sensory integration–based program was delivered for learners with autism. This program involved individual occupational therapy sessions with home programming as well as consultation with the teacher and the group home staff. The interventions were delivered by the occupational therapist and the group home staff [63]. The second individually focused intervention, focused on promoting social skills of a learner with autism through strategies (e.g., a cueing system and increasing self‐awareness and identity) while collaborating with the multimedia teacher in the planning of the intervention, as well as a group member/peer in the execution of the intervention [61]. Similarly, the third reported individually focused intervention was for a learner with autism, focused on social skills training and promoting functional independence. This intervention also included consultation services with the home economics teacher for the culinary club [57]. The last individually based intervention focused on reducing the fear of public speaking and included 15 individual sessions on relaxation and desensitization with school‐going adolescents [60].

### 3.5. Sustainability

The sustainability of occupational therapy is influenced by the human and physical resources required for service delivery, including accessibility/convenience of the intervention sessions and adequate duration of the intervention aligned with intended goals. The duration and frequency of sessions were not adequately reported in all studies. From the reported studies, the median intervention period was 11.5 weeks, with the duration of the sessions ranging from 20 to 60 min. Only 11 studies reported the frequency of the intervention. Most studies took place weekly, and nine studies explicitly reported on the materials needed to conduct the intervention. From the nine studies that reported on materials and equipment, seven studies indicated technological and therapeutic equipment such as vibrators, weighted vests, lead X‐ray aprons, plantar boxes, cell phones, multimedia rooms, projectors, floor mats, large therapy balls, video recording equipment, sound absorbing walls, and halogen lighting [62, 55, 63, 65, 59, 67, 57]. Only two studies (one on promoting social skills and one using a compensatory counting system) describe interventions where minimal equipment was needed, such as paper, a table, and a cardboard box [61, 54].

### 3.6. Cultural Norms for Participation

Only one study (about a social–cognitive intervention program for adolescents with autism) reported on cultural beliefs and practice considerations when planning and implementing an intervention program to ensure cultural relevance [58].

Occupational therapy should enable participation in all the occupational therapy domains, which include education and learning, leisure, personal and community living, play, social participation, and work [52].

Six studies focused on promoting participation in the learning domain. The majority of the intervention programs focus on the improvement of academic skills like counting [54], adaptations to the environment [55, 63], and working collaboratively with other team members ([66, 56]). Another six studies promoted social participation through interventions for mental health and well‐being [60, 68], as well as a social–cognitive intervention program and a group‐based social skills training program [58, 61, 62, 57].

Only three studies included interventions to promote participation in work, such as transition readiness interventions in the areas of prevocational skills, social skills, self‐determination, and physical fitness for work participation [64, 65, 59]. One study promoted participation in leisure occupations [63], and another study focused on independence in personal and community living (e.g., hygiene, shaving, and dressing appropriately) [57]. None of the interventions in this review promoted participation in health management–related activities or sleep and rest in schools.

#### 3.7. Spirituality

None of the studies specifically addressed the spiritual needs of adolescents at school.

#### 3.8. Results of the Most Suitable Interventions for the African Context

The proposed framework was used to evaluate the suitability of the 15 interventions discussed in the results for the African context, based on their characteristics of evidence, community‐based practice, practice approaches, sustainability, cultural norms for participation, and spirituality [19]. After the data from the 15 interventions were interpreted, it was discovered that four out of the 15 interventions showed the most promise for adaptation to the African context through the number of contextual suitability features that they share, as shown in Table 3 [58, 59, 67, 68].

**Table 3:** Common features for the suitability of interventions in Africa.

Interventions in schools	Address key challenges in Africa	Conducted in a group	Used minimal equipment	Collaborated with community members	Translated the intervention to suit the cultural norms
1. Recovery program for adolescents with substance use disorders [68]	X	X	X	X	
2. A social cognitive intervention to promote social skills in adolescents with autism [58]		X	X	X	X
3. A transition readiness service to promote work participation [59]	X	X	X	X	
4. Coping strategies to promote adolescents′ mental health in schools [67]	X	X	X	X	

## 4. Discussion

After the four most suitable interventions for the African context were identified, we realized that none addressed any dimension of spirituality. One of the models that is used in occupational therapy practice, the Canadian Model of Occupational Performance and Engagement (CMOP‐E), centers spirituality at its core and considers it the primary construct that makes an individual′s life meaningful. Through this core aspect, an individual views the world, which shapes their values, beliefs, transitions, and practices [69, 70]. Several researchers have written on spirituality and occupational therapy practice [19, 53, 70–72]. Spirituality is, therefore, a key element in caring for patients, including adolescents, and is fundamental to holistic and client‐centered approaches. [71] highlight that adolescent spirituality is intricately related to their engagement in occupations. This is further emphasized by Morris [72], who states that spirituality drives occupational performance, which is meaningful to the person. In this regard, Jones et al. [53] proposed seven dimensions of spirituality in a conceptual framework for occupational therapists to embed in their practice, namely, suffering, becoming, centeredness, transcendence, meaning, connectedness, and being. Many of these dimensions also correlate with the core features of occupational therapy group interventions, guided by Yalom [73].

The STORY program could be suitable for the African context, as it advocates for adolescents with substance use disorders at a school, which is a community‐based setting [68]. Substance abuse is often highlighted in the literature as a particular issue for adolescents from disadvantaged communities [74]. David et al. [75] argue that addressing adolescent substance abuse requires a collective effort in rural areas, where there is limited health and community infrastructure. Schools could serve as a platform for addressing issues such as substance abuse. David et al. [75] further argue that managing adolescent substance abuse should go beyond outreach or educational sessions at the school and empower the school and surrounding community with the knowledge, skills, and human resources to promote health and well‐being. Currently, there are many educational programs, community outreach initiatives, and awareness campaigns around substance abuse in African schools [76, 77]. Although this is the case, there remains a gap in managing substance abuse in schools, as school personnel are often equipped only to report the abuse rather than manage it within the school setting [76]. This STORY program intervention could be recommended as it allows for community‐based practice, where the occupational therapist cofacilitates sessions with a community health educator. Additionally, the intervention is suitable for population‐focused or group therapy, where more adolescents could be reached, compared to individual sessions.

Although spirituality was not specifically mentioned in this intervention, the dimensions of suffering and connectedness were evident in managing a group of adolescents′ substance abuse and should be incorporated into future occupational therapy interventions within the African context. Jones et al. [53] agree that suffering is a prominent feature of spirituality, and occupational therapists should address pain and loss (due to disorders/illnesses/disabilities) as part of the occupational therapy process. Connectedness (a sense of belonging created through the participation of adolescents with substance use disorder in a group) could be seen as a vehicle for spirituality [78]. Including features of occupational therapy groups (e.g., cohesiveness, installation of hope, altruism, universality, catharsis, and interpersonal learning) [73] fosters the connectedness dimension of spirituality in group therapy and a sense of belonging [78]. Occupation‐focused groups are central to occupational therapy practice and one of the preferred interventions to promote health and well‐being and participation in purposeful life roles [79].

A social cognitive intervention program for adolescents with Autism demonstrated its suitability for the African context as the only intervention that reported on cultural relevance [58]. The program was translated, and the content was checked by four experienced occupational therapists to ensure cultural relevance. Adhering to cultural norms in Africa ensures holistic and client‐centered practice, which are both central constructs in occupational therapy [19, 70]. Acknowledging the cultural norms (personal, socioeconomic, historical, and relational) that influence occupational choices and preserve occupational injustices in adolescents from disadvantaged communities could help occupational therapists reconsider their interventions to promote occupational choices and eliminate occupational injustices [80]. Similar to the first intervention, this social cognitive intervention was conducted in a group. The occupational therapist and a team member delivered the sessions, which addressed both the sustainability of the intervention (e.g., low technological use and group therapy approaches) and the practice approaches and cultural norms, by relying on the connectedness and interdependence of the group to promote social skills.

Occupational therapy transition readiness services could also be suitable to promote learners with disabilities′ participation in work [59]. Peer‐based groups were central to the intervention described by Pierce et al. [59], together with several other collaborations with staff members (e.g., school staff, principal, counselors, janitorial staff, and teachers), parents, and the community. The two key aspects that made this intervention suitable for the African context are the collaboration with the local community and the family to promote prevocational participation. Collaborating with the local community in interventions considers the important African humanistic views that a community‐based society can lead to social well‐being [22].

Collaboration with the community furthermore reinforces interdependence and reciprocal obligations between members of the community. This highlights that issues faced by adolescents should follow a bottom‐up approach where the community is included. Similarly, the involvement of the parents as collaborators in the intervention addresses the cultural norm of Ubuntu, where a person′s expression is ultimately expressed in relation to other people (the family as a whole) [70]. Additionally, the peer‐based groups address the connectedness dimension of spirituality, where the learner is connected to peers, the occupational therapist, their family, and other community members. Through this connection, well‐being is promoted [53]. Peer groups could be a valuable addition to occupational therapy interventions in Africa, as adolescents from disadvantaged communities often make choices that emphasize their connection to others, which emphasizes the importance of spirituality, interdependence, and Ubuntu [71, 74].

An intervention promoting the mental health of adolescents in schools could apply to the African context [67]. This intervention aimed to help adolescents cope better with school demands. Currently, mental health is one of the main challenges that adolescents face [81]. Teaching adolescents coping strategies for managing their mental health could be very effective in promoting educational participation. Spirituality was found to help adolescents with identity formation and occupational engagement and provide them with the strength to keep going [71]. Teaching a person coping strategies could relate to two dimensions of spirituality, namely, transcendence and becoming. Transcendence is the power of the person to view their challenges beyond the person or environment [53]. Occupational therapists could incorporate this in their therapy by including pastoral care and spiritual leaders as part of the health services team to create supportive environments to address mental health issues.

Furthermore, recognizing the person′s capacity for growth (through equipping them with skills) addressed the spirituality dimension of becoming. Learners in Africa might struggle more with their mental health due to several contextual challenges, which add additional strain to school challenges. The intervention also favored group interaction and dialogue, two key principles for occupational group therapy, namely, cohesion and connectedness. These principles create supportive environments to improve the health and well‐being of any population that struggles with mental health [78].

### 4.1. Strengths and Limitations of the Review

Our framework for evaluating interventions for suitability in the African context discovered that most of the interventions found did not apply to the African context, and none addressed spirituality. While the framework was extremely valuable for evaluating existing research, we believe it can also be used to inform the cultural adaptation of evidence‐based interventions to the African context. It is also a useful tool for occupational therapists planning and reporting intervention studies. Further occupational therapy research on interventions that can promote the participation of adolescents in African schools is needed. There are also a few limitations in this review. The review only included peer‐reviewed articles, but no other forms of literature, such as grey literature or books, were included. Additionally, only English articles were included. The literature search might have yielded different results on spirituality in the African context if non‐English articles were included, as English is not a prominent language in Africa. This review only focused on occupational therapy interventions that promote adolescent participation, which means that other information related to the roles that occupational therapists fulfill in high school was not included.

## 5. Conclusion

The proposed framework for evaluating interventions found that most interventions identified in this review did not apply to the African context, and none addressed a key contextual consideration, namely, spirituality. Four interventions that were found to be more applicable for promoting the participation of adolescents in African schools had the following common features: They were school‐based, conducted therapy in groups, used minimal equipment, collaborated with members of the community, used translators, included the family, and found collective strategies to address issues. The dimensions of spirituality should be incorporated with the common features to make interventions suitable for the African context (e.g., for group therapy, the dimension of connectedness could be incorporated to create cohesion or a sense of belonging). Occupational therapists working with adolescents in Africa should adapt their interventions to encompass all areas of participation in schools (e.g., academic and nonacademic activities) and incorporate features of culturally relevant practice rooted in African philosophies to ultimately improve participation in schools. This, in turn, could have a positive ripple effect on adolescents′ school completion, employment opportunities, and the African economy.

## Funding

No funding was received for this manuscript.

## Disclosure

The authors declare that the opinions in this submitted article are their own and not the views of Stellenbosch University.

## Conflicts of Interest

The authors declare no conflicts of interest.

## Data Availability

The data that delivered the results of this article is available from the corresponding author, Lomarie Thesnaar, upon reasonable request.
